# Spiraling Light with Magnetic Metamaterial Quarter-Wave Turbines

**DOI:** 10.1038/s41598-017-12143-7

**Published:** 2017-09-19

**Authors:** Jinwei Zeng, Ting S. Luk, Jie Gao, Xiaodong Yang

**Affiliations:** 1 Department of Mechanical and Aerospace Engineering, Missouri University of Science and Technology, Rolla, MO 65409 USA; 2 Center for Integrated Nanotechnologies, Sandia National Laboratories, Albuquerque, NM 87185 USA

## Abstract

Miniaturized quarter-wave plate devices empower spin to orbital angular momentum conversion and vector polarization formation, which serve as bridges connecting conventional optical beam and structured light. Enabling the manipulability of additional dimensions as the complex polarization and phase of light, quarter-wave plate devices are essential for exploring a plethora of applications based on orbital angular momentum or vector polarization, such as optical sensing, holography, and communication. Here we propose and demonstrate the magnetic metamaterial quarter-wave turbines at visible wavelength to produce radially and azimuthally polarized vector vortices from circularly polarized incident beam. The magnetic metamaterials function excellently as quarter-wave plates at single wavelength and maintain the quarter-wave phase retardation in broadband, while the turbine blades consist of multiple polar sections, each of which contains homogeneously oriented magnetic metamaterial gratings near azimuthal or radial directions to effectively convert circular polarization to linear polarization and induce phase shift under Pancharatnum-Berry’s phase principle. The perspective concept of multiple polar sections of magnetic metamaterials can extend to other analogous designs in the strongly coupled nanostructures to accomplish many types of light phase-polarization manipulation and structured light conversion in the desired manner.

## Introduction

Structured light, one kind of special optical beams with spatial inhomogeneous intensity, polarization or phase distributions, turns over a new leaf in the development of modern optics^1–6^. In addition to the conventional frequency and amplitude modulation, the creation of structured light including optical vortex and vector beam can as well exploit the complex phase and polarization of light, pushing the capacity of light manipulation closer to full potentials. This capability of tailoring additional dimensions on light finds a perfect synergy with the optical nanotechnology, breeding a plethora of splendid possibilities in integrated photonic circuits, quantum communication, nano-holography, and near-field sensing^7–13^.

However, it is typically difficult to simultaneously and effectively manipulate polarization and phase of light at nanoscale which is critical in ultra-compact integrated optics, because the optical anisotropy provided by naturally existing materials is generally too finite to significantly modulate light in deep-subwavelength space. The emergence of metamaterials and metasurfaces contributes a solution to this difficulty^3^. Metamaterials and metasurfaces are artificial subwavelength-structured composite materials with engineered electromagnetic properties on demand that can break nature’s limit, including artificial magnetism, tailored anisotropy, negative index of refraction, near zero permittivity or index, and thus can provide sufficient control over light as required^14–22^.

A typical approach for simultaneous manipulation of light polarization and phase is by transmitting incident beam through an anisotropic medium, where the two originally independent physical quantities are coupled and connected. The relationship between the polarization and phase variation of the transmitted beam can be described by the space-domain geometric phase—‘the Pancharatnam-Berry phase’^23,24^, discovered by the pioneer scientists^25–29^. Based on this principle, the anisotropic optical element, termed as the Pancharatnam-Berry phase Optical Element (PBOE), can be used to induce the designated phase and polarization manipulations. To date, a plenty of complex light manipulation including the generation of scalar and vector vortices have been enabled by the smartly designed metamaterial or metasurface based PBOEs in special orientations and patterns including nano-rods, nano-gratings, V-shape antennas, split-ring antennas, and multilayer cavities^9,30–40^.

Among many kinds of PBOEs, here we are particularly interested in the optical element that can induce quarter-wave retardation between the two orthogonal linear polarization axes, acting as a quarter-wave plate (QWP). Such element has the unique property that can convert circular polarization (CP) into linear polarization (LP) with the polarization direction depending on the QWP orientation. Structured polarization and phase then can be created by the spatially inhomogeneous array of the QWP elements under the PBOE principle. In this work, we propose and demonstrate the magnetic metamaterial quarter-wave turbines at visible wavelength to twist circularly polarized incident beam into radially and azimuthally polarized vector vortices. The magnetic metamaterial elements based on metal-dielectric-metal three-layer structures are designed to utilize both the electric and magnetic field of light and realize QWP functionality at the designated wavelength and maintain the quarter-wave retardation in broadband, inducing the broadband circular-to-linear polarization conversion. The turbine blades are made of multiple polar sections of the homogeneously oriented magnetic metamaterial QWP gratings with the fast axis of the QWP element in approximately +/−45° to the azimuthal angle. The constructed metamaterial quarter-wave turbines will transform the incident CP to vector polarization with helical phase front of charge 1. Furthermore, the vector polarization distributions of the produced vortices can be conveniently tuned azimuthally or radially by simply switching the spin of the incident CP beam or the turbine rotation direction.

## Results

### Design and characterization of magnetic metamaterial QWP gratings

**Figure 1:**
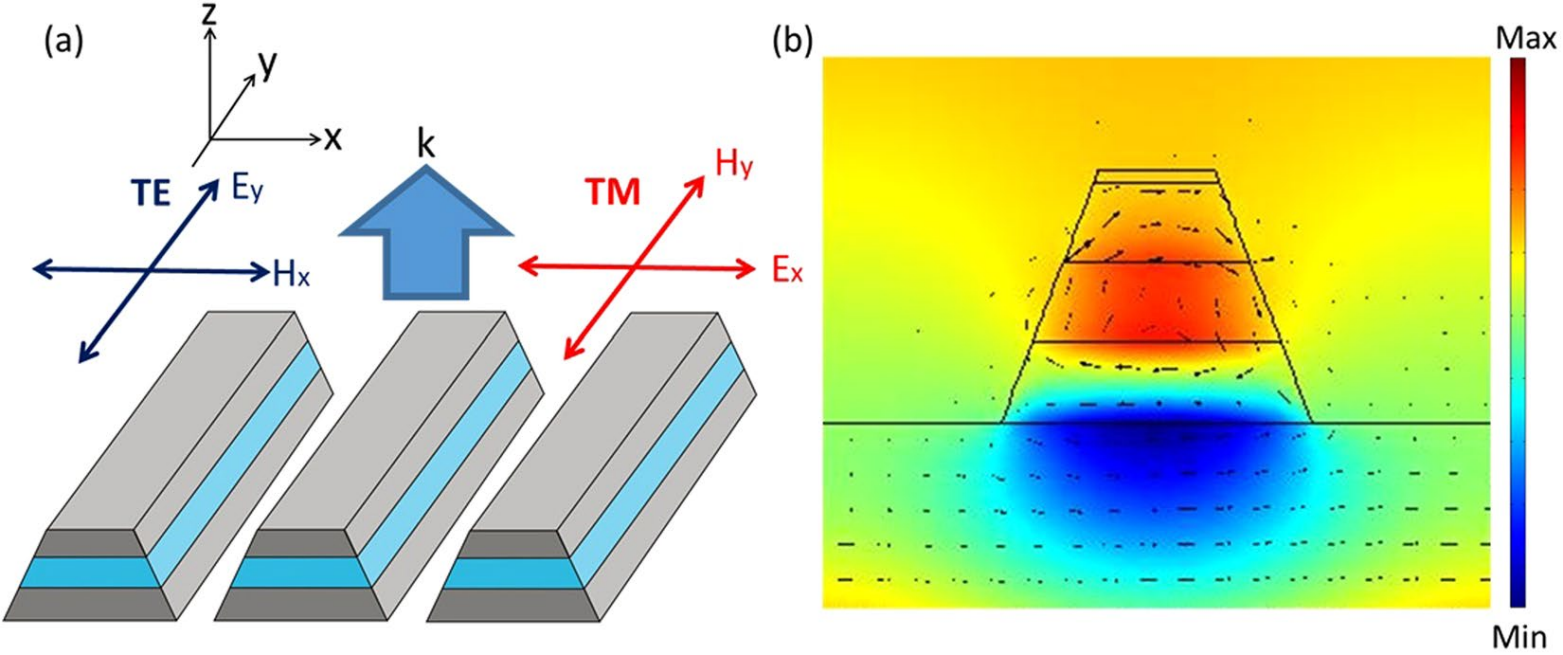
(**a**) The schematic of magnetic metamaterial gratings in a metal-dielectric-metal three-layer structure. (**b**) The simulated magnetic field (color map) and electric displacement (black arrows) on magnetic resonance under TM polarization.

The homogeneous magnetic metamaterial gratings are designed, fabricated, and characterized to confirm its functionality as a good QWP, which will serve as the fundamental building blocks for constructing the spatial inhomogeneous magnetic metamaterial quarter-wave turbines. As shown in Fig. 1, a typical magnetic metamaterial grating structure contains a dielectric spacer sandwiched by two metal stripes in a subwavelength periodic pattern. The physical mechanism of such structure has been well studied by the previous literatures^38,41–44^. Magnetic resonances can be excited under transverse-magnetic (TM) illumination in this structure, where the magnetic field of the incident beam is parallel with the metal stripes. The incident magnetic field produces a circulating displacement current loop encircling the dielectric spacer with anti-parallel surface currents between the pair of metal stripes, which then excites a magnetic dipole moment normal to the current loop and parallel to the metal stripes. Magnetic resonance can then be excited causing a possibly negative effective permeability and an abrupt transmission phase variation across the resonance wavelength. Under transverse-electric (TE) polarized illumination where the electric field is parallel with the metal stripes, on the contrary, this structure resembles a diluted metal without supportable optical resonances, and has a generally flat transmission phase spectrum. As a result, the magnetic metamaterial gratings are able to manipulate both electric and magnetic fields of light at the same time, which can simultaneously control the transmission amplitude and phase in the orthogonal polarizations. Such control over the two orthogonal polarizations is important here. A perfect waveplate requires simultaneous phase anisotropy and amplitude isotropy. However, while the magnetic resonance induces the required phase retardation, it also reduces the amplitude transmission under TM polarization. This weakened amplitude transmission under TM polarization can then be balanced with the suppressed transmission by the grating effect under TE polarization to satisfy the amplitude isotropy requirement.

**Figure 2:**
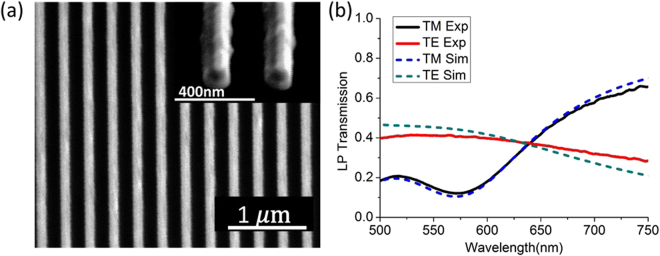
(**a**) The SEM images of the top-view and cross-section of the fabricated magnetic metamaterial grating sample. (**b**) The measured and simulated transmission spectra of the magnetic metamaterial grating sample.

In this work, we design and fabricate the magnetic metamaterial grating samples to realize nearly ideal QWP devices operating near the wavelength of HeNe laser at 633 nm, with the excited magnetic resonance around 575 nm. The design principle is based on the physics of the magnetic metamaterials: the resonance wavelength is mainly determined by the width of the cavity and independent of the grating period; the phase retardation between TE and TM can be controlled by varying the thickness of each layer; the amplitude balance between TE and TM can be achieved by changing the grating period to modify the filling fraction of this structure. Eventually, by scanning these structural parameters of the structure in simulation, the optimized parameter set can be achieved for giving the best performance as QWP and such parameter set will be used in the sample fabrication. The final fabricated structure is as follows. The three-layer Ag-SiO_2_-Ag structure is deposited on glass slide by the sputtering method at the deposition rates of 0.4 Å/sec and 0.2 Å/sec for Ag and SiO_2_ layers, respectively, which has a thickness of 40 nm for each layer, together with an additional 5 nm thick SiO_2_ surface protection layer on top. The magnetic metamaterial grating pattern is then milled into the Ag-SiO_2_-Ag multilayer by focus ion beam (FIB) lithography with the optimized parameters of grating period as 300 nm and bottom metal stripe width as 140 nm. In all of the following experimental and modeling configurations, the samples are always placed so that the incident beam enters from the glass side to the multilayer side. The SEM image of the fabricated magnetic metamaterial grating sample is shown in Fig. 2(a).

Numerical simulation of the proposed magnetic metamaterial QWP grating is performed with the finite element method COMSOL Multiphysics. The final geometries of the modeled structure, including a slightly tilted side-wall of the grating cross section, are obtained from the SEM image of the fabricated structure shown in Fig. 2(a). The Ag and SiO_2_ permittivities are acquired from the variable angle spectroscopic ellipsometry. We adjusted the loss of the Ag in the simulation so that the simulated transmission spectrum fits the experimental transmission spectrum, according to the previous study on the similar structure^44^ for the purpose of acquiring a more accurate estimation of the transmission phase. The simulated and measured transmission spectra of this structure are shown in Fig. 2(b). It shows the distinctive feature of the modeled magnetic resonance under TM polarization is around 575 nm, where the circulating displacement current loop encircles around the magnetic dipole as shown in Fig. 1(b). The magnetic resonance introduce a transmission minimum and an abrupt quarter-wave phase shift across the resonance under TM polarization, and the created phase difference is preserved stably above the resonance wavelength. This quarter-wave retardation and the proximity of equal TE and TM transmissions near 633 nm demonstrate the realization of a decent QWP with its fast-axis along TE direction.

**Figure 3:**
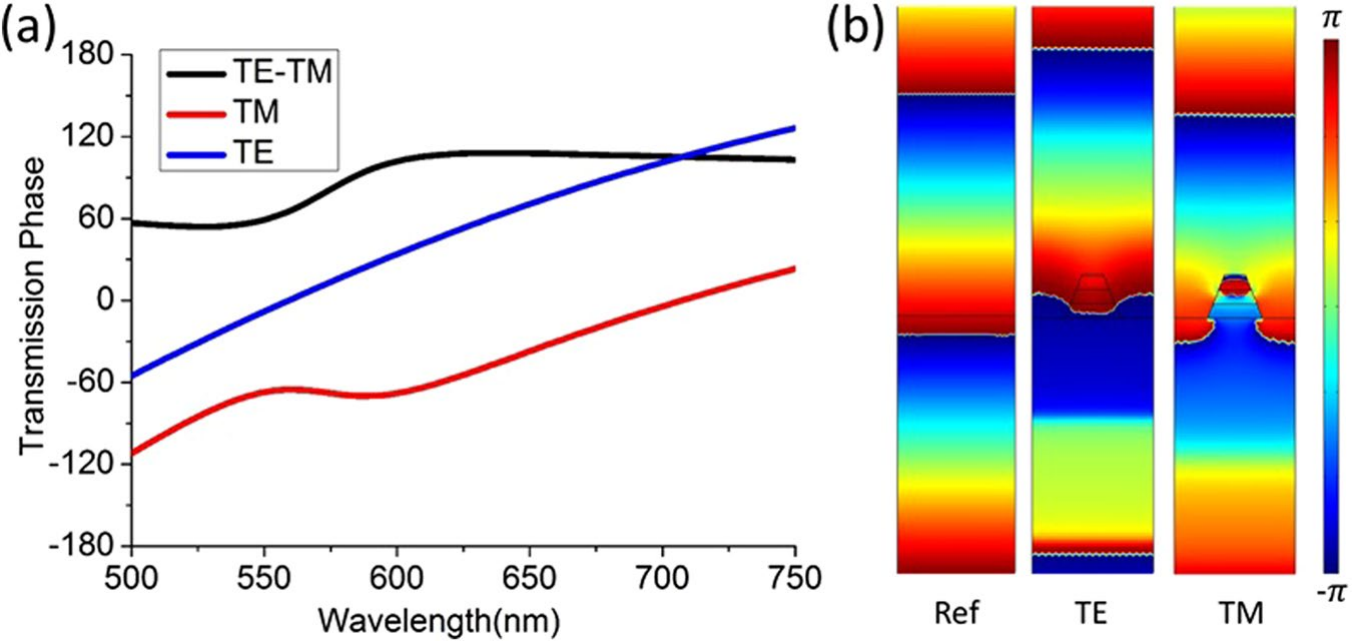
(**a**) The simulated transmission phase spectra of the magnetic metamaterial grating. (**b**) The simulated spatial field phase distribution of the grating structure under TE and TM polarizations comparing with the air reference.

From Fig. 3(a), it is interesting to observe that the phase retardation between TE and TM polarizations is nearly a constant around 90° after the magnetic resonance wavelength. Since there are no more resonances above 633 nm, this constant phase retardation is expected to be broadband extending to the longer wavelength side of 750 nm, until the relative thickness of this structure as compared to wavelength further decreases and can not support the required phase retardation. The nearly constant phase retardation should be attributed to the magnetic resonance supported in the longitudinal grating cavity, since such phenomenon does not exhibit in the structure of single metal layer. The semi-infinitely broadband constant quarter-wave phase retardation is a very desirable property, as this structure can serve as a broadband CP-to-LP converter. However, as the amplitude proximity is only satisfied in a limited bandwidth around 633 nm, the QWP function is specified in this particular narrow wavelength region.

Next, the performance of circular-to-linear polarization conversion by the magnetic metamaterial QWP grating is quantitatively characterized. It is emphasized that, as CP and LP are not mutually orthogonal, we cannot define energy-based polarization conversion efficiency between them. Instead, we illustrate such polarization conversion by inspecting the degree of linear polarization (DoLP) and the angle of linear polarization (AoLP) of the transmitted beam under CP incidence in both experiment and simulation. The DoLP is defined by the polarization Stokes parameters expressed in Equation (1), and the AoLP represents for the orientation angle of the dominant linear polarization component^17,20,45^.

1 $$DoLP=\frac{\sqrt{{s}_{1}^{2}+{s}_{2}^{2}}}{{s}_{0}}$$

**Figure 4:**
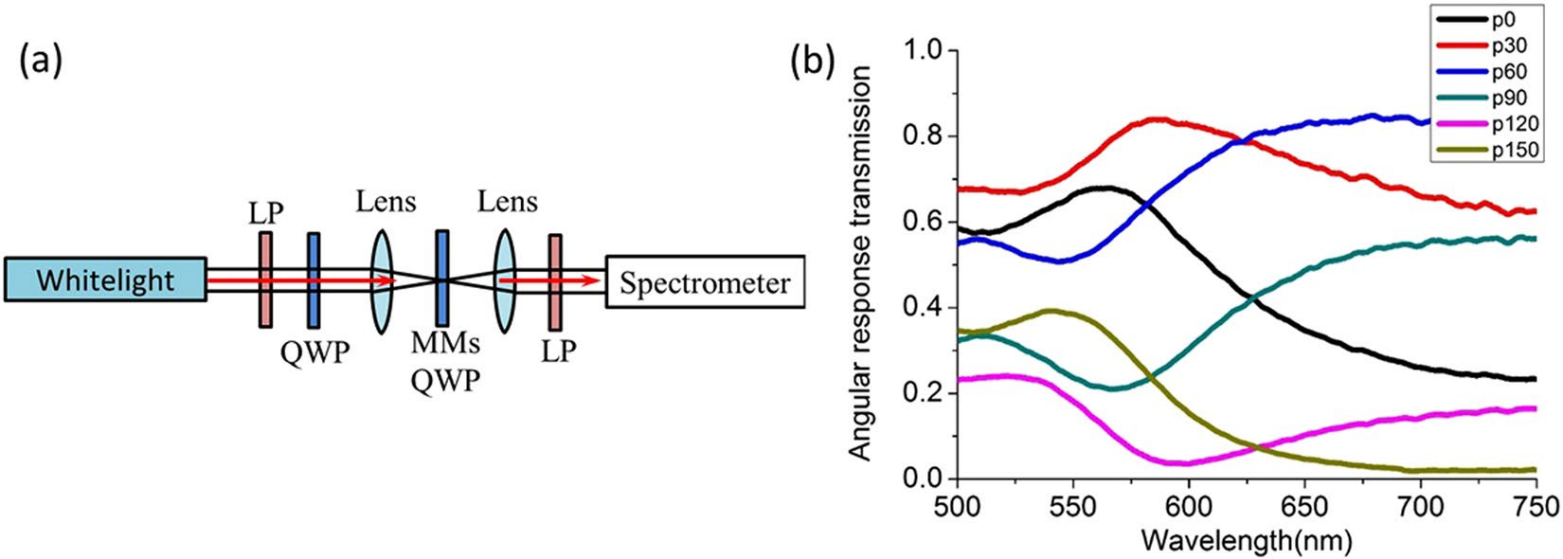
(**a**) The schematic of the experimental setup for polarization analysis. (**b**) The measured polarization analysis spectra with different rotation angle of the linear polarization analyzer. LP: linear polarizer, MMs: metamaterials.

Where *s*_0_ *s*_1_ and *s*_2_ are the corresponding Stokes parameters. Here, by considering the magnetic metamaterial gratings as effective media, the transmitted beam from CP incidence will have a general elliptical polarization. Its Stokes parameters, in experiment, can be obtained based on the polarization ellipse, which is characterized by the classical polarization analysis experiment^17,46^ as shown in Fig. 4. In this experiment, a CP beam is first created by passing the white light beam from a halogen lamp through a linear polarizer and a quarter-wave plate which has a 45° tilted angle between their axes, then the beam is normally focused through the magnetic metamaterial grating sample with the stripes oriented vertically. Next, the transmitted beam from the sample passes through a rotating linear polarization analyzer, and the spectra of the final beam with angular-response are measured by a spectrometer. The transmission spectra are normalized by the intensity of the beam before passing the analyzer. The corresponding polarization ellipse parameters at each wavelength can be retrieved by a sinusoidal fitting function shown in Equation (2).

2 $$T(\psi )={a}^{2}co{s}^{2}(\psi -\theta )+{b}^{2}si{n}^{2}(\psi -\theta )$$

where *ψ* represent for the angle of polarization analyzer, *a* and *b* represent for the long and short radius of the polarization ellipse, and *θ* represent for the tilted angle of the ellipse (AoLP). Then the Stokes parameters can be calculated by the following equations related to the electric field components *E*_*a*_ and *E*_*b*_^47^.

3 $$\begin{array}{c}{s}_{0}={E}_{a}^{2}+{E}_{b}^{2}\\ {s}_{1}=({E}_{a}^{2}-{E}_{b}^{2})cos(2\theta )\\ {s}_{2}=({E}_{a}^{2}-{E}_{b}^{2})sin(2\theta )\end{array}$$

**Figure 5:**
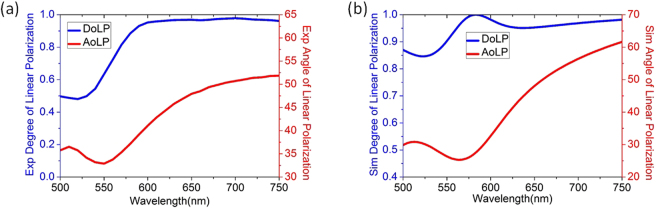
(**a**,**b**) The DoLP and AoLP retrieved from experiment and simulation, respectively.

Based on the analysis from Equations (1)–(3), the DoLP and AoLP in experiment are derived and plotted in Fig. 5(a).

In simulation, the magnetic metamaterial grating unitcell under the same CP illumination is modeled, from which the normalized field transmittance can be acquired. Then the Stokes parameters and the tilted angle of the polarization ellipse are calculated from Equation (4) below^47^.

4 $$\begin{array}{c}{s}_{0}={|{E}_{x}|}^{2}+{|{E}_{y}|}^{2}\\ {s}_{1}={|{E}_{x}|}^{2}-{|{E}_{y}|}^{2}\\ {s}_{2}=2Re({E}_{x}{E}_{y}^{\ast })\\ \theta =\frac{1}{2}{\rm{atan}}(\frac{2{E}_{x}{E}_{y}}{{E}_{x}^{2}-{E}_{y}^{2}}\,\cos (\delta ))\end{array}$$

Figure 5(b) plots the simulated DoLP and AoLP, which exhibits a decent agreement with the experimental results. As shown in Fig. 5, the DoLP and AoLP spectra demonstrate the magnetic metamaterial grating sample operates as an excellent QWP near 633 nm, where the transmitted beam has the DoLP and AoLP close to unity and 45°, respectively.

It is remarkable that the function of QWP can be realized by many different metamaterial and metasurface structures as illustrated by the previous research^17,19–21^. Besides, other works study optical HWP element using dielectric nanostructures with near-unity polarization (spin) conversion efficiency and minimum optical loss^48–50^. Analogous ideas could also be applied to make dielectric QWP element with similarly excellent performance. Comparing to these works, the most significant feature of our current structure is such magnetic metamaterial grating maintains the quarter-wave retardation in a broadband wavelength range in direct transmission mode, thus it can produce a highly linearly polarized beam with DoLP larger than 95%, above the magnetic resonance wavelength in broadband. Further, our structure has a relatively thin thickness as one quarter of wavelength, and it is convenient to fabricate as a one-dimensional grating structure, in contrast to the reported dielectric-post waveplate which has a thickness close to the wavelength and the thickness is larger than the period. These merits can be an important advantage at least for now, as fabricating a structure with larger longitudinal-to-transverse aspect ratio is generally more difficult especially for devices operating in visible frequency range.

### Vector vortex generation by magnetic metamaterial quarter-wave turbines

**Figure 6:**
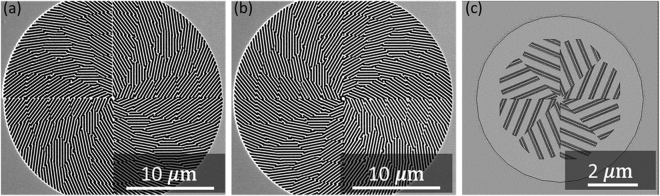
(**a**,**b**) The SEM image of the fabricated magnetic metamaterial quarter-wave turbines with counter-clockwise rotation and clockwise rotation, respectively. (**c**) The simulated counter-clockwise rotation turbine structure.

By combining the magnetic metamaterial QWP gratings with certain orientations as the building blocks, the spatial inhomogeneous quarter-wave turbines are constructed with each grating section as one turbine blade, as shown in the SEM images of the fabricated samples in Fig. 6. These metamaterial quarter-wave turbines are designed to create vector vortices from CP incident beam. The whole turbine pattern is segmented into multiple polar sections, which individually has the uniformly oriented magnetic metamaterial QWP grating with its fast-axis in approximate +/−45° to the radial-direction for constructing the counter-clockwise rotation turbine or the clockwise rotation turbine, respectively. Therefore, the QWP gratings within each section will convert the incident CP beam into LP beam with its polarization direction in +/−45° to the QWP fast-axis, making an overall radial or azimuthal polarization for the final transmitted beam through the entire turbine. According to the PBOE principle, a rotation distribution of CP-to-LP polarization conversion element in a full 2*π* around an origin will produce a charge +1 or −1 optical vortex from CP incidence^27,29,38^. Here, the generation of optical vortices by the turbines is broadband due to the broadband CP-to-LP conversion of the magnetic metamaterial grating, although outside the optimized QWP wavelength region near 633 nm the polarization state of the transmitted beam will not be purely radial or azimuthal but their hybrid.

The mathematical derivation of vector vortex generation by the ideal quarter-wave turbines is as follows. First, the CP incident beam can be expressed in the cylindrical vector coordinates. Taking the left-handed circular polarized (LHC) beam for example, the electric field is

5 $$\begin{array}{rcl}{E}_{LHC} & = & P(r)\frac{\sqrt{2}}{2}({{\boldsymbol{e}}}_{{\boldsymbol{x}}}+i{{\boldsymbol{e}}}_{{\boldsymbol{y}}})\\  & = & P(r)\frac{\sqrt{2}}{2}((\cos (\phi ){{\boldsymbol{e}}}_{{\boldsymbol{r}}}-\,\sin (\phi ){{\boldsymbol{e}}}_{\phi })\\  &  & +i(\sin (\phi ){{\boldsymbol{e}}}_{{\boldsymbol{r}}}+\,\cos (\phi ){{\boldsymbol{e}}}_{\phi }))\\  & = & P(r)\frac{\sqrt{2}}{2}{e}^{i\phi }({{\boldsymbol{e}}}_{{\boldsymbol{r}}}+i{{\boldsymbol{e}}}_{\phi })\end{array}$$

where $P(r)$ is the spatial amplitude profile, *φ* is the azimuthal angle, ***e***_***r***_ and ***e***_***φ***_ are unit vectors in the radial and azimuthal direction, respectively. Then, define a new coordinate system with two axes that have a +/−45° rotation to the radial direction, in which the pair unit vectors are defined as ${{\boldsymbol{e}}}_{{\boldsymbol{r}}+{45}^{\circ }}$ and ${{\boldsymbol{e}}}_{{\boldsymbol{r}}-{45}^{\circ }}$ respectively, as ${{\boldsymbol{e}}}_{{\boldsymbol{r}}+{45}^{\circ }}=\frac{\sqrt{2}}{2}({{\boldsymbol{e}}}_{{\boldsymbol{r}}}-{{\boldsymbol{e}}}_{\phi })$, and ${{\boldsymbol{e}}}_{{\boldsymbol{r}}-{45}^{\circ }}=\frac{\sqrt{2}}{2}({{\boldsymbol{e}}}_{{\boldsymbol{r}}}+{{\boldsymbol{e}}}_{\phi })$, so that

6 $$\begin{array}{c}{{\boldsymbol{e}}}_{{\boldsymbol{r}}}=\frac{\sqrt{2}}{2}({{\boldsymbol{e}}}_{{\boldsymbol{r}}+{45}^{\circ }}+{{\boldsymbol{e}}}_{r-{45}^{\circ }}),\\ {{\boldsymbol{e}}}_{\phi }=\frac{\sqrt{2}}{2}({{\boldsymbol{e}}}_{{\boldsymbol{r}}+{45}^{\circ }}-{{\boldsymbol{e}}}_{r-{45}^{\circ }})\end{array}$$

Then the LHC beam can be expressed in the corresponding *r* + 45° and *r* − 45° coordinates as:

7 $$\begin{array}{rcl}{E}_{LHC} & = & P(r)\frac{\sqrt{2}}{2}{e}^{i\phi }(\frac{\sqrt{2}}{2}({{\boldsymbol{e}}}_{{\boldsymbol{r}}{\boldsymbol{+}}4{{\bf{5}}}^{\circ }}+{{\boldsymbol{e}}}_{{\boldsymbol{r}}-{45}^{\circ }})+i\frac{\sqrt{2}}{2}({{\boldsymbol{e}}}_{{\boldsymbol{r}}{\boldsymbol{+}}4{{\bf{5}}}^{\circ }}-{{\boldsymbol{e}}}_{r-{45}^{\circ }}))\\  & = & \frac{1}{2}P(r){e}^{i\phi }((1+i){{\boldsymbol{e}}}_{{\boldsymbol{r}}{\boldsymbol{+}}4{{\bf{5}}}^{\circ }}+(1-i){{\boldsymbol{e}}}_{{\boldsymbol{r}}{\boldsymbol{-}}4{{\bf{5}}}^{\circ }})\end{array}$$

After the LHC beam passing through the clockwise rotation turbine, which is treated as the QWP with its fast axis at −45° to the radial direction, the transmitted beam can be expressed as:

8 $$\begin{array}{rcl}{E}_{out} & = & \frac{1}{2}\,P(r){e}^{i\phi }t\,((1+i){{\boldsymbol{e}}}_{{\boldsymbol{r}}+{\bf{4}}{{\bf{5}}}^{\circ }}+(1-i){{\boldsymbol{e}}}_{r-{\bf{4}}{{\bf{5}}}^{\circ }})(\begin{array}{c}1\\ i\end{array})\\  & = & \frac{\sqrt{2}}{2}(1+i)t{e}^{i\phi }{{\boldsymbol{e}}}_{r}\end{array}$$

where *t* is the amplitude transmittance of the QWP element. This expression shows that the transmitted beam has the radial polarization with a charge +1 orbital angular momentum (OAM). The circumstances with an arbitrary combination of incident spin and turbine rotation direction can be calculated accordingly with the following conclusions for the phase and polarization manipulations. First, LHC incident beam will always generate charge +1 OAM, while RHC incident beam will always generate charge −1 OAM, where spin-to-orbital angular momentum conservation is satisfied. Second, radial polarization will be produced for LHC input with clockwise rotation turbine or RHC input with counter-clockwise rotation turbine; while azimuthal polarization will be produced for LHC input with counter-clockwise rotation turbine and RHC input with clockwise rotation turbine.

Concerning the general method of vector vortex generation by polarization conversion under the PBOE principle, it is noteworthy that the linear polarizer element in special patterns such as concentric rings can also achieve similar functionality as illustrated by some previous works^31,38,51^. However, the QWP element, as a comparison, has two distinguished merits. First, the QWP element exploits the complete incident field while the linear polarizer element instantaneously blocks half of the field, which grants the QWP element to provide higher energy efficiency. Second, the vector polarization produced by the QWP element can be conveniently switched from radial to azimuthal by inversing the incident spin or switching the QWP fast-axis orientation.

**Figure 7:**
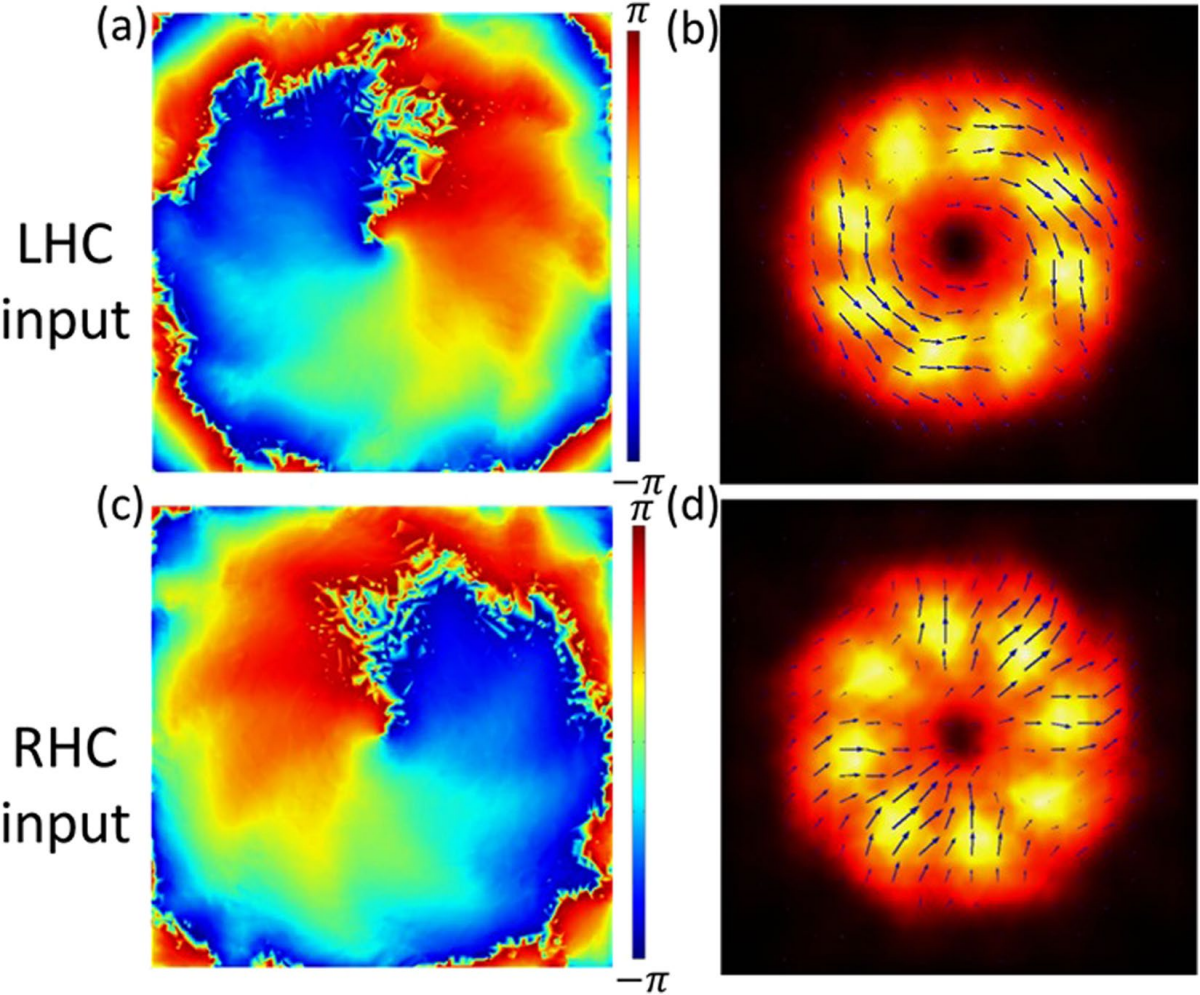
(**a**,**c**) The simulated phase distribution of the generated vector vortices at near-field with LHC and RHC incident beam transmitted through the counter-clockwise rotation turbine, creating charge +1 and −1 OAM, respectively. (**b**,**d**) The simulated intensity profile (color map) and polarization distribution (black arrows) of the generated vector vortices with LHC and RHC incident beam through the counter-clockwise rotation turbine, producing azimuthal or radial polarization profiles, respectively.

Next, the 3D simulation of the magnetic metamaterial quarter-wave turbine is performed by using the COMSOL Multiphysics. The simulated structure is shown in Fig. 6(c), where only the inner-most eight polar sections of the counter-clockwise rotation turbine are included. The structural and material parameters of each grating unitcell in the 3D simulation are acquired from those used in the previous simulation for the magnetic metamaterial grating structure, and the illumination field has been set as either LHC or RHC beam. The phase profile and polarization distribution of the transmitted beam at near-field are calculated and plotted in Fig. 7, which clearly indicate the vector vortex generation as expected.

**Figure 8:**
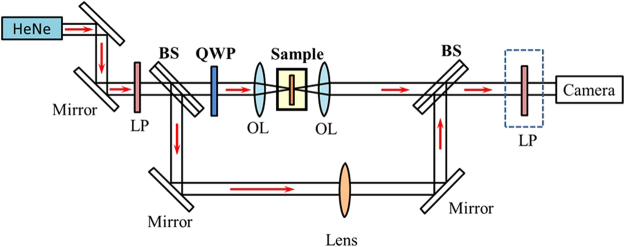
The schematic of characterization experimental setup for the transmission imaging, spherical wave interferometry and polarization analysis of the generated vector vortices through the magnetic metamaterial quarter-wave turbine sample. OL: objective lens, BS: beam splitter, LP: linear polarizer.

Based on the fabrication parameters of the homogeneous magnetic metamaterial gratings, the metamaterial quarter-wave turbine samples with both counter-clockwise rotation and clockwise rotation are fabricated and characterized. The total fabrication area for each turbine sample is a disk with a 24 *μ*m-diameter, which is divided into four quadrants during the FIB milling. There are slightly stitching errors at the boundary between adjacent quadrants. The SEM images of the fabricated counter-clockwise rotation turbine and clockwise rotation turbine are shown in Fig. 6. The characterization experimental setup is shown in Fig. 8, where a CP incident beam output from a 633 nm HeNe laser through a linear polarizer and a QWP is normally focused on the turbine sample. Then the transmitted beam is re-collimated and characterized by spherical wave interferometry and polarization analysis for studying the output OAM and polarization distribution.

**Figure 9:**
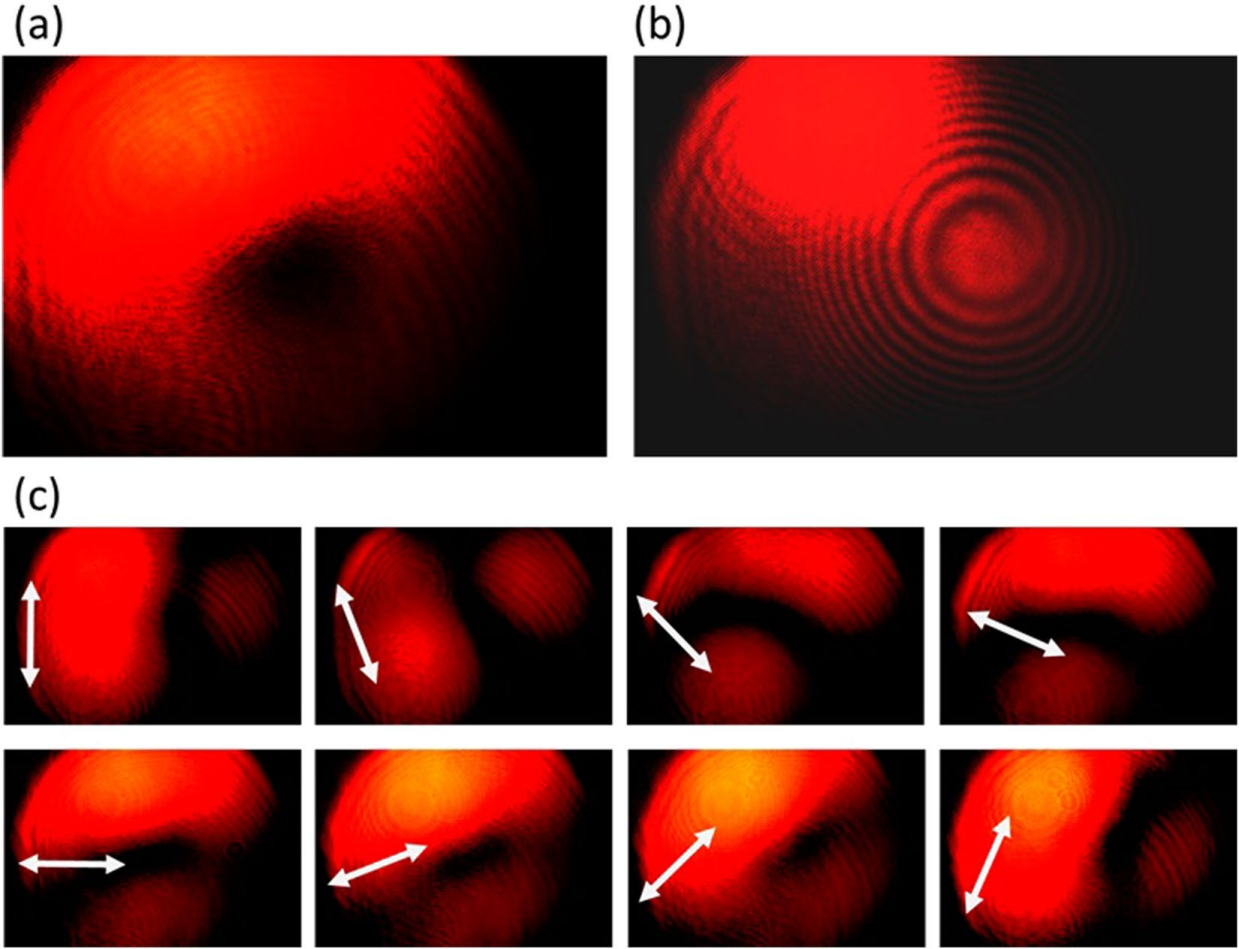
The generated azimuthally polarized vector vortex with charge +1 from the counter-clockwise rotation turbine under LHC input. (**a**),(**b**) The direct transmission images and the interference pattern of the vector vortex, respectively. (**c**) The transmission images after the vector vortex passing a rotating linear polarization analyzer, where the white arrows represent for the orientation angles of the polarization analyzer from 0 to 7π/8.

**Figure 10:**
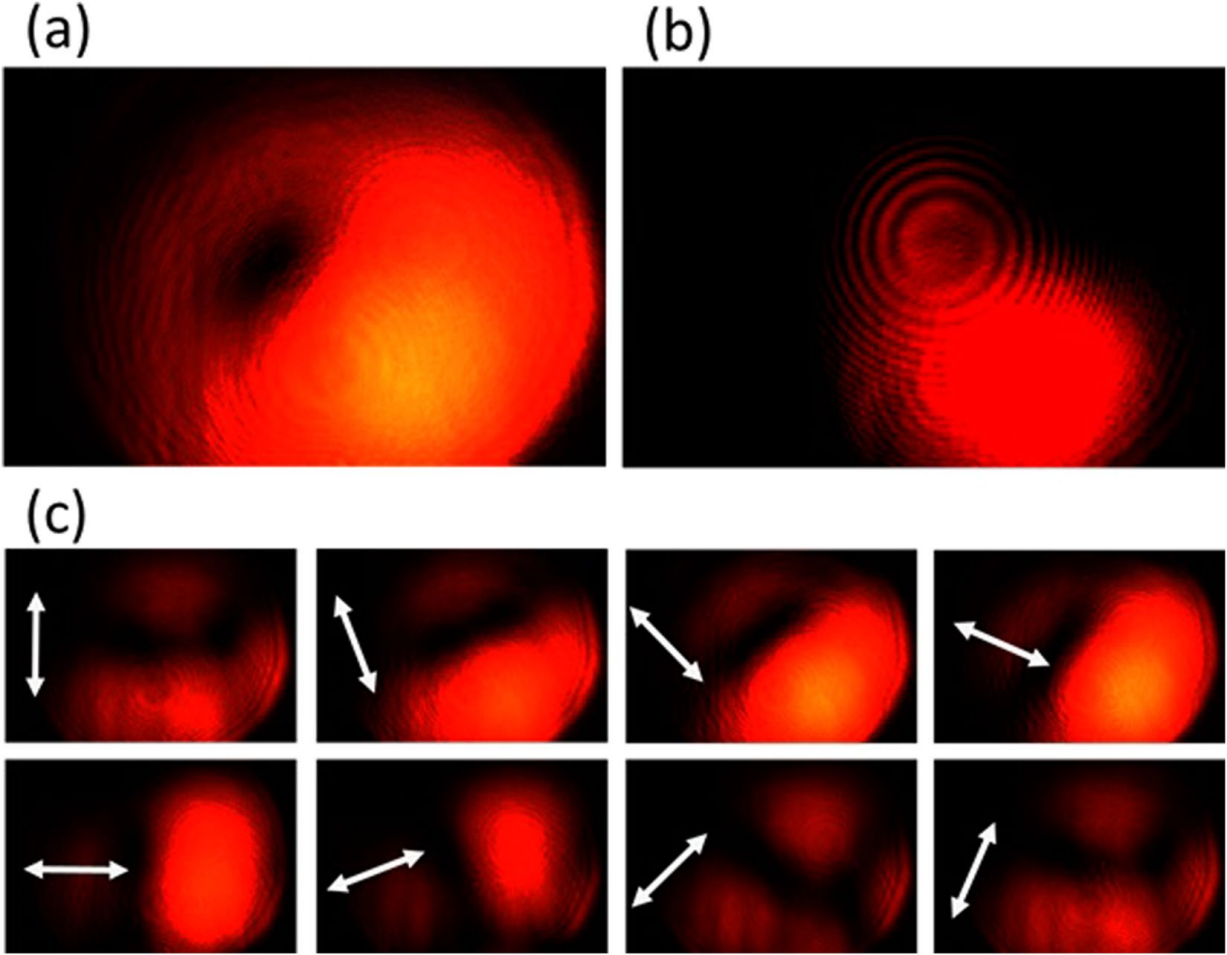
The generated radially polarized vector vortex with charge −1 from the counter-clockwise rotation turbine under RHC input. (**a**),(**b**) The direct transmission images and the interference pattern of the vector vortex, respectively. (**c**) The transmission images after the vector vortex passing a rotating linear polarization analyzer.

**Figure 11:**
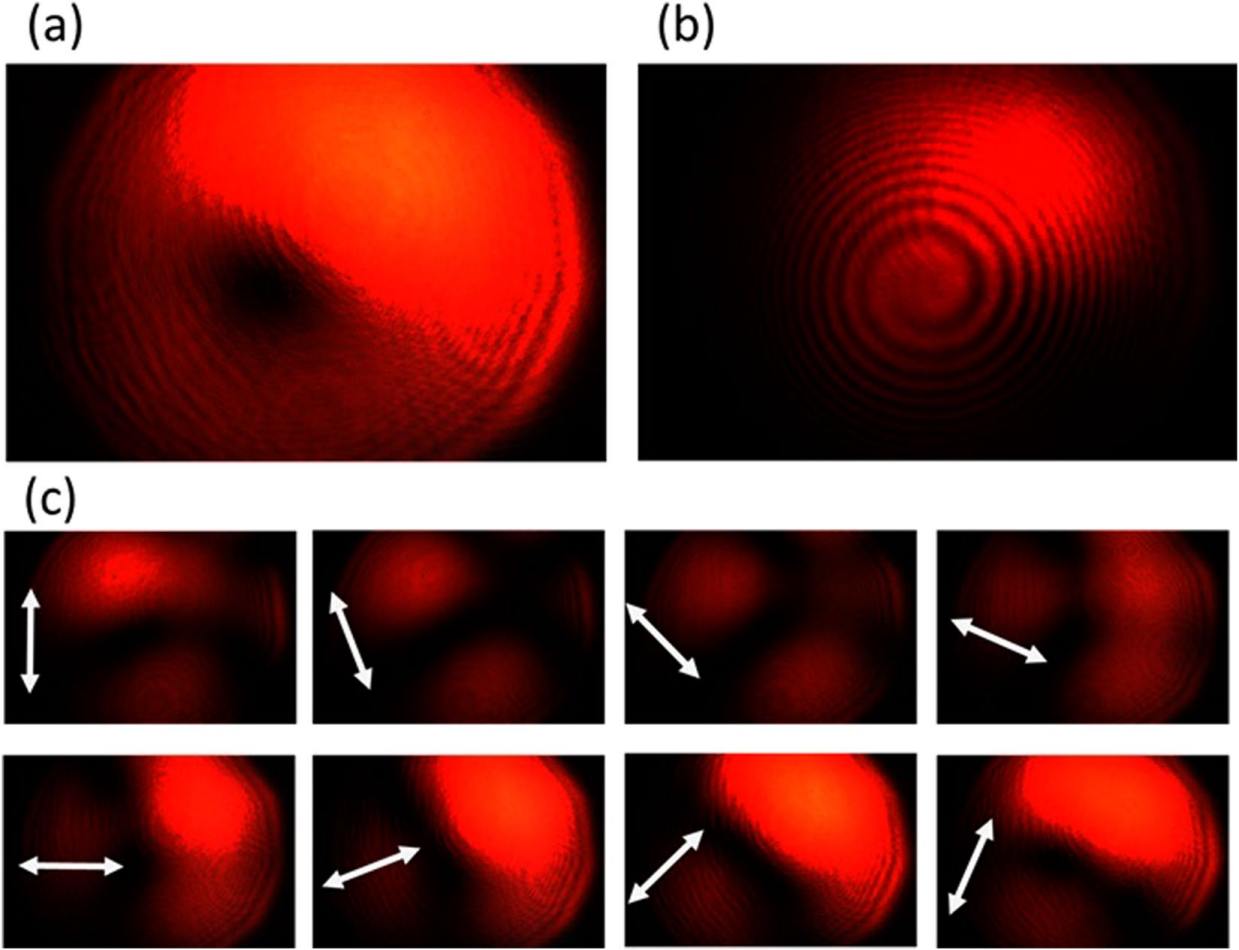
The generated radially polarized vector vortex with charge +1 from the clockwise rotation turbine under LHC input. (**a**),(**b**) The direct transmission images and the interference pattern of the vector vortex, respectively. (**c**) The transmission images after the vector vortex passing a rotating linear polarization analyzer.

**Figure 12:**
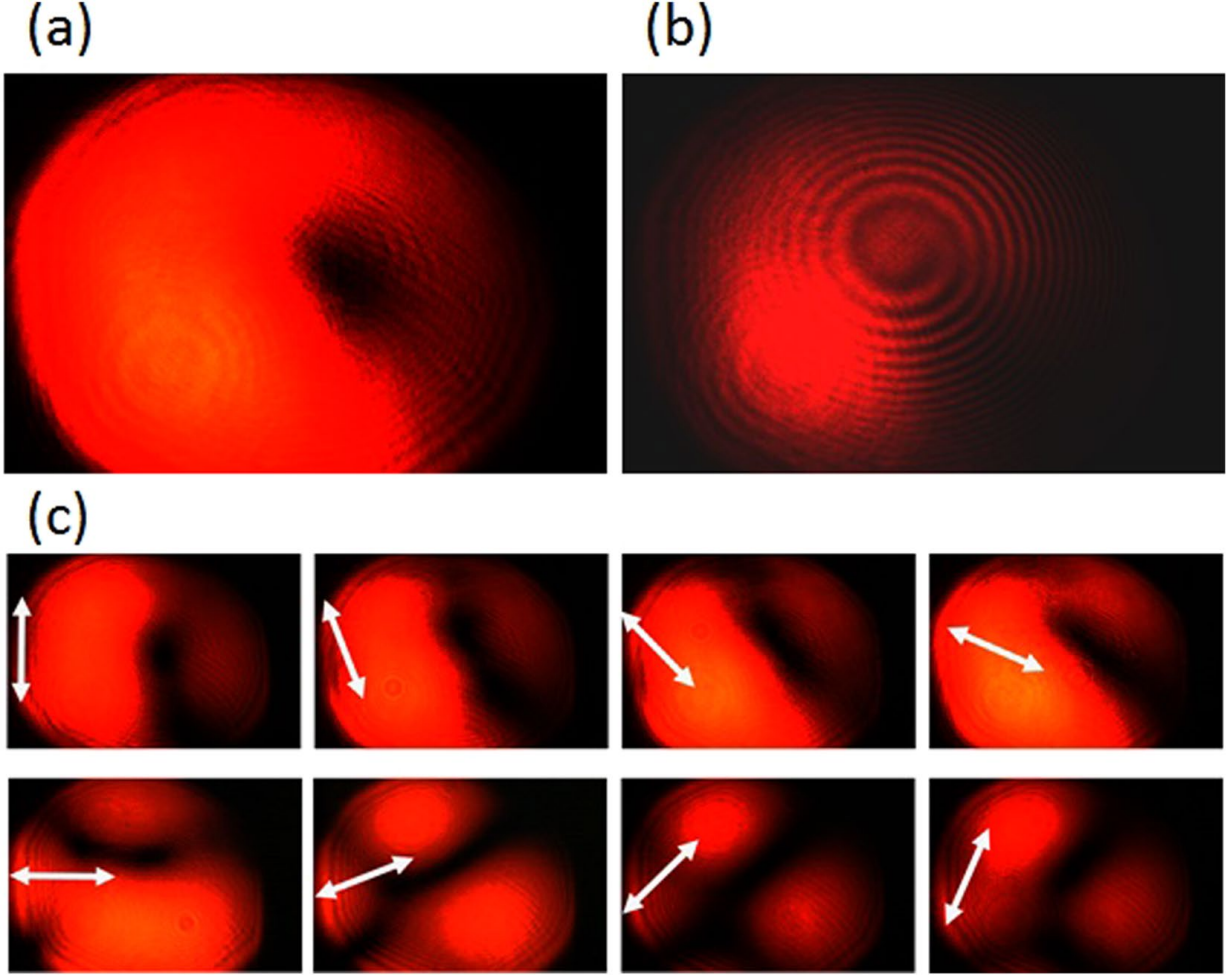
The generated azimuthally polarized vector vortex with charge −1 from the clockwise rotation turbine under RHC input. (**a**,**b**) The direct transmission images and the interference pattern of the vector vortex, respectively. (**c**) The transmission images after the vector vortex passing a rotating linear polarization analyzer.

The experimental results are summarized in Figs 9–12, for all the combinations of incident spin of LHC/RHC and turbine counter-clockwise/clockwise rotation direction. The interference patterns in all cases exhibit single spiral fringes, which has counter-clockwise orientation for LHC incidence or clockwise orientation for RHC incidence, indicating either charge +1 or −1 OAM is obtained in the output vector vortex beam. The transmission images after the linear polarization analyzer always exhibit a two-lobe shape, which rotates together with the rotation angle of the polarization analyzer. The azimuthal or radial polarizations are signified when the darkline between the two lobes is in parallel or perpendicular with the polarization analyzer direction, respectively. The polarization analysis results present that azimuthally polarized vector vortices are generated when LHC (or RHC) incident beam transmits through counter-clockwise (or clockwise) rotation turbine, while radially polarized vector vortices are produced when LHC (or RHC) incident beam transmits through clockwise (or counter-clockwise) rotation turbine. These experimental observations further confirm the theoretic and modeling results explained previously.

## Discussions

Lastly, we discuss some important principles and outcomes about our proposed concept and demonstrated results. It is noteworthy that the cross-coupling between neighboring PBOEs has a substantial impact on the overall PBOE array design. For weakly coupled optical elements in which the transmission is independent on their periodicity orientation, such like low filling-fraction plasmonic or dielectric nano-antennas, desired phase manipulation can be induced by simply rotating their functional part (antenna) without rotating periodicity^34,37,40^. Spatial inhomogeneous PBOE array based on such weakly coupled PBOEs is most convenient to the designs with excellent flexibility and accuracy. The magnetic metamaterials studied here, on the other hand, are the strongly coupled grating structures, and we must rotate the whole unitcell in order to effectively control the transmitted phase from the PBOE principle. Thus, except for some very special patterns such as concentric rings, it is almost impossible to make spatial inhomogeneous patterns while perfectly maintaining the periodicity for strongly coupled structures. This is a setback. However, on the other hand, strongly coupled structures usually have stronger interaction over the incident beam, thus can realize designated function as waveplate or polarizer with thinner device thickness. For example, the magnetic metamaterial QWPs here realize the QWP function with the total thickness less than one quarter of the wavelength, comparing to the weakly coupled dielectric slab structure which has the thickness close to one wavelength^48–50^. A thinner optical thickness makes such structure a closer approximation as “optical surface” with more robust tolerance to oblique incidence, very desirable in many optical applications. Furthermore, as mentioned before, our magnetic metamaterial grating structure has a nearly 50% filling fraction and an approximate 1:2.5 height-to-period aspect ratio which makes it convenient to fabricate.

We developed the polar sectioned grating array to address the periodicity rotating difficulty. Each polar section contains uniform grating unitcells whose periodicity is perfectly preserved. But the boundaries between adjacent sections bring slight error to light manipulation. Naturally the denser the sections, the more accurate the phase and polarization manipulation can be achieved. However, each section should contain enough number of grating periods to make it physically effective for the incident beam. Eventually we design each polar section to contain about 10 grating periods.

Another noteworthy point is that the intensity profiles of the generated vortices exhibit a certain degree of asymmetry. There may be some different contributing factors, among which the most significant reason, in our understanding, is due to the stitching error in the fabrication process. The geometrical errors in the section boundaries from design and the stitching error from fabrication together induce diffractions of the transmitted beam, causing a split from the eigenmode of the optical vortex and eventually produce non-integer OAM and hybrid polarization states in the output. Previous studies suggest that mixed modes of optical vortices are not stable^1^, and are susceptible to split into composite singularities in far field, which can induce an asymmetry intensity profile^52,53^.

The stitching error between the adjacent quadrants shown in Fig. 6(a,b) is due to the alignment shift in the FIB tool when we fabricate the quadrants sequentially. Naturally, such error could be eliminated if the complete fabrication pattern can be loaded into the FIB tool as one bitmap. Previous work shows highly symmetric vortex can be generated via a similar structure from a perfect fabrication by a powerful tool^54^. This intensity asymmetry in the current work, on the other hand, can be cleaned by applying suitable optical filter such like few-mode fiber in post-processes^55^.

Finally, we emphasis that the magnetic metamaterial QWPs in the +/−45° turbine blades presented here is just one example of using multiple polar sections to enable spatial inhomogeneous light manipulation under PBOE principle. This particular structure can only produce +1/−1-charge radially or azimuthally polarized beam; however, through a different unitcell and orientation inside the polar sections, arbitrary light phase and polarization control can be achieved based on the optically-thin strongly coupled nanostructures.

## Conclusions

In summary, we design, fabricate and characterize spatial inhomogeneous magnetic metamaterial quarter-wave turbines to create radially and azimuthally polarized vector vortices from circularly polarized incident beam at visible wavelength. The metamaterial turbines are constructed from the sectioned magnetic metamaterial gratings oriented along the specified angles exhibiting excellent QWP feature in the assigned wavelength and broadband efficient CP-to-LP conversion response. Following the PBOE principle, the metamaterial quarter-wave turbines are able to convert the CP incident beam into vector vortex beam with spin-dependent charge +1 or −1 OAM. Moreover, the switch between the azimuthally or radially polarized vector vortex beam is realized by simply reversing the incident spin or flipping the turbine rotation direction. Our demonstrated magnetic metamaterial quarter-wave turbine device is compact, efficient and tunable. It can be integrated into the future photonic and optoelectronic circuits for structured beam conversion, wavefront shaping, particle trapping and communications. Our proposed device will also serve as an inspirational example for exploring many other on-chip optical devices in complex polarization manipulation and phase shaping.
